# COVID-19 Cases, Hospitalizations, and Deaths Among American Indian or Alaska Native Persons — Alaska, 2020–2021

**DOI:** 10.15585/mmwr.mm7122a2

**Published:** 2022-06-03

**Authors:** Lowrie A. Ward, Kelsey P. Black, Carla L. Britton, Megan L. Tompkins, Ellen M. Provost

**Affiliations:** ^1^Alaska Native Epidemiology Center, Alaska Native Tribal Health Consortium, Anchorage, Alaska; ^2^Section of Epidemiology, Division of Public Health, Alaska Department of Health and Social Services.

American Indian or Alaska Native (AI/AN) persons across the United States face substantial health disparities, including a disproportionately higher incidence of COVID-19 (*1*,*2*). AI/AN persons living in Alaska also face serious health and health care challenges, including access to care because 90% of the state’s land area is inaccessible by road (*3*), and approximately one half of the state’s AI/AN population (AI/AN race alone or in combination with another race) live in remote rural areas (*4*). To examine the extent of COVID-19–associated disparities among AI/AN persons living in Alaska, a retrospective analysis of COVID-19 cases reported to the Alaska Department of Health and Social Services (AKDHSS) during March 12, 2020–December 31, 2021, was conducted. The age-adjusted COVID-19 incidence among AI/AN persons was 26,583 per 100,000 standard population, approximately twice the rate among White persons living in Alaska (11,935). The age-adjusted COVID-19–associated hospitalization rate among AI/AN persons was 742 per 100,000, nearly three times the rate among White persons (273) (rate ratio [RR] = 2.72). The age-adjusted COVID-19–related mortality rate among AI/AN persons was 297 per 100,000, approximately three times that among White persons (104; RR = 2.86). Culturally competent public health efforts that are designed in collaboration with AI/AN persons and communities, including support for vaccination and other proven COVID-19 prevention strategies, are critical to reducing COVID-19–associated disparities among AI/AN persons in Alaska.

A retrospective analysis was conducted of COVID-19 incidence, and associated hospitalizations and deaths in Alaska reported to AKDHSS Section of Epidemiology during March 12, 2020–December 31, 2021.* Data analyzed consisted of a limited data set received through a data sharing agreement with AKDHSS Section of Epidemiology. COVID-19 cases were defined in accordance with CDC’s National Notifiable Disease Surveillance System.^†^ COVID-19–associated hospitalizations were defined as hospital admissions of COVID-19 patients because of severity and complications of COVID-19. Deaths were determined with death certificate audits and included decedents who had received a diagnosis of laboratory-confirmed COVID-19, as well as deaths that were likely COVID-19–related based on clinical and epidemiologic criteria as defined by CDC, with no confirmatory laboratory testing. Groups assessed by race and ethnicity included AI/AN race (alone or in combination with other races), White race alone, other races (including those not reporting AI/AN heritage who were Asian, Black or African American, Native Hawaiian or other Pacific Islander, or multiple races), and unknown race. The unknown race category included persons for whom race was not recorded, or for whom race was still under investigation.

Population proportions and age-adjusted COVID-19 case, hospitalization, and mortality rates were calculated to account for differences in underlying population age distributions. Age was aggregated into 10-year age groups. The AI/AN population in Alaska is younger than the overall state population because of higher birth rates, and because the size of the population born during 1946–1964 was small (*3*). Rates were calculated by age group and race, using the direct method standardized to the U.S. 2000 standard population and the most recent Alaska population estimates (*4*). Corresponding 95% CIs were calculated based on the gamma distribution (*5*). Bivariate analyses used Fisher’s exact test given the lack of normality of the underlying data; p-values <0.05 were considered statistically significant. RRs were calculated using age-adjusted rates, with White persons as the referent group; corresponding 95% CIs that excluded 1 were considered statistically significant. COVID-19 vaccination data by race were not compatibly categorized. To assess the effect of records with unknown race on observed disparities in COVID-19 outcomes, a sensitivity analysis was performed by recalculating the RR, categorizing all those with unknown race as White persons. All analyses were conducted using R (version 1.2.5001; RStudio). This activity was reviewed by AKDHSS, the Alaska Native Tribal Health Consortium Non-Research Review Group, and CDC and was conducted consistent with applicable federal law and CDC policy.^§^

During March 12, 2020–December 31, 2021, a total of 159,043 COVID-19 cases were reported in Alaska. Cases in nonresidents (5,717 [3.6%]) and those in Alaska residents reported out of state (1,064 [0.7%]) were excluded from further analysis; the final analytic data set included 152,262 in-state resident cases. AI/AN persons (alone or in combination with another race), White persons, and persons of other races accounted for 39,338 (25.8%), 55,415 (36.4%), and 19,615 (12.9%) persons with COVID-19, respectively; race was unknown for 37,894 (24.9%) patients (Table 1). Among persons with COVID-19, those who were AI/AN were younger (70.1% aged <40 years) compared with those of all other races (59.0% aged <40 years) and more were female (52.5%) compared with those of all other races (47.9%).

**TABLE 1:** COVID-19 incidence and outcomes by race, sex, and age group — Alaska, March 12, 2020–December 31, 2021

Characteristic	No. (%)				
	AI/AN*	White^†^	Other^§^	Unknown^¶^	Total
	(n = 39,338)	(n = 55,415)	(n = 19,615)	(n = 37,894)	(N = 152,262)
**Sex**					
Female	20,637 (52.5)	26,405 (47.6)	9,730 (49.6)	17,928 (47.3)	74,700 (49.1)
Male	18,679 (47.5)	28,883 (52.1)	9,833 (50.1)	19,657 (51.9)	77,052 (50.6)
Unknown	22 (0.1)	127 (0.2)	52 (0.3)	309 (0.8)	510 (0.3)
**Age group, yrs**					
<10	6,704 (17.0)	4,567 (8.2)	1,798 (9.2)	3,844 (10.1)	16,913 (11.1)
10–19	7,261 (18.5)	6,657 (12.0)	2,575 (13.1)	5,381 (14.2)	21,874 (14.4)
20–29	6,887 (17.5)	9,602 (17.3)	4,167 (21.2)	6,705 (17.7)	27,361 (18.0)
30–39	6,734 (17.1)	10,282 (18.6)	3,844 (19.6)	7,164 (18.9)	28,024 (18.4)
40–49	4,121 (10.5)	7,742 (14.0)	2,602 (13.3)	5,556 (14.7)	20,021 (13.1)
50–59	3,531 (9.0)	7,212 (13.0)	2,307 (11.8)	4,522 (11.9)	17,572 (11.5)
60–69	2,496 (6.3)	5,566 (10.0)	1,529 (7.8)	3,106 (8.2)	12,697 (8.3)
70–79	1,147 (2.9)	2,670 (4.8)	587 (3.0)	1,142 (3.0)	5,546 (3.6)
≥80	457 (1.2)	1,117 (2.0)	206 (1.1)	474 (1.3)	2,254 (1.5)
**Hospitalizations**	823 (2.1)	1,438 (2.6)	675 (3.4)	359 (0.9)	3,295 (2.2)
**Deaths**	289 (0.7)	521 (0.9)	159 (0.8)	51 (0.1)	1,020 (0.7)

**Abbreviation:** AI/AN = American Indian or Alaska Native.

* AI/AN race alone or in combination with other races.

**^†^** White race alone.

^§^ Included Asian, Black or African American, Native Hawaiian or other Pacific Islander race and ethnicities, or multiple races not including AI/AN heritage.

^¶^ Race was not recorded and cases are still under investigation.

Among 3,295 (2.2%) hospitalized COVID-19 patients, 823 (25.0%) were AI/AN persons, 1,438 (43.6%) were White persons, and 675 (20.5%) were persons of other races. Overall, 1,020 (0.7%) Alaska COVID-19 patients died; 289 (28.3%) deaths occurred among AI/AN persons, 521 (51.1%) among White persons, and 159 (15.6%) among persons of other races.

The age-adjusted COVID-19 incidence was 26,583 per 100,000 persons among AI/AN persons compared with 11,935 among White persons (RR = 2.23) (Table 2). The age-adjusted COVID-19–associated hospitalization rate was 742 per 100,000 among AI/AN persons compared with 273 among White persons (RR = 2.72), and the age adjusted COVID-19 mortality rate was 297 per 100,000 among AI/AN persons compared with 104 among White persons (RR = 2.86). Among persons of other races, the age-adjusted COVID-19 incidence was 18,268 per 100,000 persons, the age-adjusted COVID-19–associated hospitalization rate was 775 per 100,000, and the age-adjusted mortality rate was 209 per 100,000.

**TABLE 2:** COVID-19 incidence, hospitalization, and death rates, by race* — Alaska, March 12, 2020–December 31, 2021

Cases and outcomes	No.	Incidence^†^ (95% CI)		Rate ratio (95% CI)
		Unadjusted	Age-adjusted	
**Cases**				
AI/AN^§^	39,338	26,564 (26,303–26,828)	26,583 (26,310–26,859)	2.23 (2.18–2.28)
White	55,415	11,731 (11,633–11,829)	11,935 (11,834–12,037)	Ref
Other	19,615	18,090 (17,832–18,345)	18,268 (18,004–18,534)	1.53 (1.5–1.57)
**Hospitalizations**				
AI/AN^§^	823	556 (518–595)	742 (689–798)	2.72 (2.36–3.13)
White	1,438	304 (289–321)	273 (258–288)	Ref
Other	675	623 (576–671)	775 (714–840)	2.84 (2.47–3.27)
**Deaths**				
AI/AN^§^	289	195 (173–219)	297 (262–336)	2.86 (2.28–3.61)
White	521	110 (101–120)	104 (94–113)	Ref
Other	159	147 (125–171)	209 (176–247)	2.02 (1.58–2.57)

**Abbreviations:** AI/AN = American Indian or Alaska Native; Ref = referent group.

* Among persons of known race. White = White race alone; Other = Asian, Black or African American, Native Hawaiian or other Pacific Islander race, or multiple races not including AI/AN heritage.

^†^ Cases per 100,000 persons.

^§^ AI/AN persons alone or in combination with other races.

A sensitivity analysis that categorized persons of unknown race as White persons resulted in an RR of 1.31 (95% CI = 1.29–1.33) for COVID-19 cases in AI/AN persons compared with White persons and persons of unknown race. The RR of COVID-19–associated hospitalizations among AI/AN persons compared with White persons and those of unknown race was 2.18 (95% CI = 1.91–2.48), and of COVID-19–related deaths was 2.62 (95% CI = 2.11–3.29) for AI/AN persons compared with White persons and persons of unknown race.

## Discussion

In Alaska, AI/AN persons had significantly higher adjusted rates of COVID-19, COVID-19–associated hospitalization, and COVID-19–related deaths compared with rates among White persons. Overall, although making up 20.3% of the state’s population (*4*), AI/AN persons accounted for approximately one quarter of Alaska’s COVID-19 cases and COVID-19–associated hospitalizations, and approximately 28% of COVID-19–related deaths. These findings are similar to those of other studies (*2*,*6*) during the COVID-19 pandemic, demonstrating a continued disproportionate impact of COVID-19 outcomes on AI/AN persons. These results are also consistent with the experience of AI/AN persons living in Alaska during the influenza A(H1N1) pandemic of 2009 (*7*), as well as the general experience of AI/AN persons in the United States with pneumonia and influenza (*8*).

The observed disparities among AI/AN persons could be the result of multiple factors. Historical trauma and structural racism negatively affect the health and well-being of AI/AN persons (*9*). In addition, living in rural and remote areas can result in increased health risks and decreased access to and use of health care (*10*). Despite additional health care needs, obtaining medical services is often challenging in rural communities. In Alaska, health care services are provided using a hub and spoke model, with community and regional clinics connected with small critical access hospitals in larger hub communities.^¶^ Tertiary care hospitals that provide advanced care are only located in urban areas (Anchorage/Matanuska-Susitna, Fairbanks, and Juneau), and travel to these facilities can be expensive, difficult, and time-consuming, resulting in less frequent health care visits for many persons.

Several actions can be taken to help achieve health equity among AI/AN persons in Alaska. Public health professionals should continue to work with tribal health organizations in Alaska to provide culturally competent and regionally required health interventions. Existing health promotion initiatives in AI/AN communities, including those related to COVID-19, can be integrated with cultural interventions to enhance relevance and respect the knowledge and wisdom of these communities as experts on their own needs (*9*). Lessons learned from AI/AN communities can also be collected and shared; COVID-19 vaccination rates vary by community, with some predominantly AI/AN communities having very high numbers of eligible residents being vaccinated.**

The findings in this study are subject to at least three limitations. First, race was unknown or still under investigation for 24.9% of cases, 11% of hospitalizations, and 5% of deaths. The extent of this exclusion on the observed disparities is unknown; however, the Tribal Health System, which is available to AI/AN persons in Alaska, more consistently documents and reports race data than do other reporting organizations and facilities. Findings from a sensitivity analysis indicate that disparities in COVID-19–associated hospitalization and COVID-19–related deaths also occurred when patients with unknown race were categorized as White persons. Second, data were restricted to the state of Alaska, and thus might not be generalizable to other AI/AN persons in the United States. Finally, the analysis was conducted on data available from cases reported in 2020 and 2021. Inclusion of additional data after further investigation of case, hospitalization, and mortality status could impact the magnitude of the observed estimates.

AI/AN persons in Alaska are at increased risk for COVID-19 illness, COVID-19–associated hospitalization, and COVID-19–related death compared with other races. Culturally competent public health efforts that are designed in collaboration with AI/AN persons and communities, including support for vaccination and other proven COVID-19 prevention or treatment strategies, are critical to reducing COVID-19–associated disparities among AI/AN persons in Alaska.

**Summary**

**What is already known about this topic?**

American Indian or Alaska Native (AI/AN) persons across the United States face substantial health disparities, including a disproportionate incidence of COVID-19 illness.

**What is added by this report?**

AI/AN persons living in Alaska are at increased risk for COVID-19 illness, COVID-19–associated hospitalization, and COVID-19–related death compared with White persons living in Alaska. Rate ratios for age-adjusted case, hospitalization, and mortality rates for AI/AN persons compared with White persons in 2020 and 2021 were 2.2, 2.7, and 2.9, respectively.

**What are the implications for public health practice?**

Culturally competent public health efforts designed in collaboration with AI/AN persons and communities, including support for vaccination and other proven COVID-19 prevention strategies, are critical to reducing COVID-19–associated disparities among AI/AN persons in Alaska.

## Footnotes

* Data were retrieved on February 2, 2022.

†
https://ndc.services.cdc.gov/case-definitions/coronavirus-disease-2019-2021/

§ 45 C.F.R. part 46; 21 C.F.R. part 56; 42 U.S.C. Sect. 241(d), 5 U.S.C. Sect. 552a, 44 U.S.C. Sect. 3501 et seq.

¶
https://dhss.alaska.gov/dph/Emergency/Documents/healthcare/publications/AlaskaStateHealthCareEnvironment.pdf

** https://experience.arcgis.com/experience/a7e8be4adbe740a1bad1393894ee4075/ (Accessed February 2, 2022).
